# Clinical Review, and Hospital Reports

**Published:** 1840

**Authors:** 


### Clinical Review.

### WESTMINSTER OPHTHALMIC HOSPITAL.

#### Cure of Squinting by the Division of one of the Recti Muscles.

In our experience we remember nothing like the rage for dividing one of the recti muscles. The surgical world is brimfull of it. The first question that one surgeon asks another when they meet, is "Have you performed this operation?" Any one who has not done it is really an object of compassion. He stands alone, and people pity him or wonder at him. Poor Dr. Franz, who introduced the operation into London, was soon buried amongst the shoal of operators, and if he has since lifted his feeble voice, it has been lost amidst the din.

As the proceeding may now be viewed as an established one of approved benefit, of wide utility, and of such easy execution, as to admit of any surgeon's effecting it; as every one, in fact, who can operate at all ought to be able to operate here, we shall bring together *some* of the facts or the suggestions that have lately appeared in connexion with it.

I. The Half-yearly Report laid before the Governors of the Royal Westminster Ophthalmic Hospital, on the 25th July, 1840. By Charles W. G. Guthrie, Jun.

We are greatly pleased to see Mr. Charles Guthrie appear upon the scene. He is a young surgeon of much promise, and bids fair to succeed to the honourable position so justly held by his able father. We wish him the success which we cannot doubt he will obtain.

Mr. Guthrie observes :—

"The operation was proposed by Dr. Stromeyer of Hanover, but was first practised by Dr. Dieffenbach of Berlin, in January last: and Mr. Pyper, a student, who had been recommended to the notice of the learned Professor by my father, and had assisted Dr. Dieffenbach in several of his operations, caused the necessary instruments to be made for him on his return to London in March, and pointed out the different steps of the operation as he had seen them performed. The first operation was done on the 18th of April, but the instruments have since been considerably modified, and the operation has been rendered more simple, so that it may be readily accomplished in less than one minute, with the greatest precision and safety on an adult, offering no resistance; I have done it in a great many instances in half a minute, and it has even been done by Mr. Guthrie in a few seconds by one introduction of the small curved knife. But this method is not safe, for the ball of the eye may be injured by any irregular motion of the patient, which did occur in one unruly boy, but neither in this nor in any other instance has any evil result, or more than slight inflammation, followed the operation in the eighty-four cases in which it has been done in this hospital or in private life, nor in others in which I have assisted; and in no case has it failed in overcoming the immediate evil."

The degree of power, says Mr. G. which the person possesses over the eyes is occasionally very doubtful on the first inspection of them; both eyes squinting in turn, or together, and it is only after a little delay that the really defective eye can be clearly ascertained; whilst in others the motions of the eye are altogether so irregular, and so little under control, that it becomes doubtful which muscle should be divided, or whether it would be proper to attempt any operation at all. In most instances, persons have seen double for two or three days, or even for a longer time after the operation, but this gradually subsided in every instance, save one, as the axis of the affected eye began to correspond with that of the other. In the exception a slight cast can be perceived on a careful examination, so slight, however, as scarcely to be remarked. In one case the sound eye turns in more than the eye which has been operated upon, but vision is single. In four cases, the eyes seemed disposed to turn inwards again for a few days, but from this they eventually recovered. In one case it has been necessary to repeat the operation—in two a new pupil has been made —in almost all, vision has been in a greater or less degree gradually improved, although the amendment of the appearance has been in some the essential point gained. In a few cases, the sight of each eye appears to be equally good. In one case the eye turned, and continues turned a little outwards after the operation.

*Operation on Children.*—When a child under eight or ten years of age, or an unruly one who may be older, of which we have had one instance at seventeen, is the subject for operation, it must be secured in the same way as in the operation for cataract, by being laid on a narrow table and covered by a strong sheet, which ought to be tied firmly underneath. The head is to be placed on a pillow, and steadily held by a spare assistant, whilst another, if necessary, further secures the body; for children of four or five, are sometimes so strong, and resist so much after the operation has been begun, as to render it very difficult to complete it, unless perfectly mastered, when they become comparatively quiet. In the first case of this kind, the muscle was not divided, and the operation had to be repeated. The youngest child Mr. G. has operated on was two years and three months old, the two next youngest were four years old.

*Operation on Adults.*—"When a patient is an adult he may be placed in a high-backed chair with the head reclined. An assistant raises the upper lid in the gentlest manner, with the ordinary silver elevator used for the same purpose in cases of soft cataract in children, holding it with one hand nearly perpendicularly to the forehead, and desiring the patient to turn the eye *outwards* in a case of squinting *inwards*, fixes a strong double hook with the other hand into the tunica albuginea, through the conjunctival membrane a little distance from the cornea, in the middle line of the eye, or what is pedantically called its equator. The points of the hook are short that they may not enter, or scarcely enter, the sclerotic coat, but they are long enough to pierce the tunica albuginea, for if they only penetrate the conjunctival membrane, this slips, and they raise it from the ball and cause its elevation near the cornea, which delays the cure. The assistant must also take care that the elevator duly raises the lid and gently confines it against the edge of the orbit in such manner that it may not slip, nor allow the upper conjunctive fold or part of the internal portion of the lid to bulge out below it, which will be apt to occur unless it is carefully prevented, by holding the elevator in the manner directed. On the right eye the elevator is to be held in the left hand, the hook with the right, and vice versa. The operator (or an assistant) having depressed the under eyelid with the fore or second finger of the left hand, directs the assistant to draw the eyeball gently outwards with the hook, until the semilunar fold of the conjunctival membrane begins to yield to the traction, when it should be held perfectly steady on the middle line, the centre of the pupil being directly under the shaft of the hook. He then makes an incision nearly equidistant from the hook, and the edge of the semilunar fold, through the conjunctival and the cellular membrane which may intervene between it and the tendon of the rectus muscle, directly upwards and downwards and inwards towards the orbit. Some surgeons use a small straight knife for this purpose, some a pair of scissors, in which case the conjunctival membrane may be raised by a pair of eye forceps at the semilunar valve, and the fold thus formed between it and the hook divided, but I generally cut through this part at once with a small curved knife, which is introduced under the conjunctiva, from below the line of the hook, the point being brought out upwards through the membrane, or with scissors convex on the lower edge, and cutting sharply up to the points which are blunt. The incision is to be enlarged if necessary to at least three-eighths of an inch in length, which exposes the tendon of the muscle going to be implanted into the outer or sclerotic coat, and which is made more distinct by the point of the knife or scissors whichever are used, or by the blunt end of the small, flat, curved and slightly grooved director, for which the knife is to be exchanged, and any blood which flows is to be taken up with a small piece of sponge. If the incision is made too close to the semilunar valve, the subjacent cellular membrane frequently becomes infiltrated with blood, and prevents the muscle from being seen, which does not occur when the incision is made at a greater distance from it, but this need not delay the operation. The curved director is now to be introduced by a gentle steady motion beneath the tendon, carrying it inwards rather deeply through the cellular and fatty membranes, so as to be passed under the muscular, as well as the tendinous part, which causes the eye to roll a little INWARDS: and which should not be prevented by holding the hook too firmly. The point is then to be raised by depressing the handle, when it will appear at the upper part of the incision, having the muscle on its grooved surface; and this elevation of the tendinous attachment of the muscle turns the eyeball OUTWARDS, so that the hook is, in fact, no longer necessary, and may be dispensed with after the first incision is made through the conjunctival membrane: and in very determined persons it may be dispensed with altogether. The director being now held in the left hand over the lower eyelid, the curved knife is to be run along the slightly marked groove of the director, and the muscle becoming tendinous is divided. The operation may be done throughout with the same pair of blunt ended curved scissors with which it was begun, the lower convex limb being gradually introduced under the muscle in the same manner and with the same precautions as the director. When the muscle is fully divided there is usually a small spurt of blood, which I consider a satisfactory sign, but which soon ceases to flow, and the eye is generally observed to turn a little outwards, but not always forcibly. The elevator should then be removed, when the upper eyelid falls, and the eye is to be sponged clean. The patient should now be desired to open the eye by raising the lid, and to turn the eye inwards; if he cannot do this the operation has been completed, but if he can do it in the slightest degree, the muscle or its lateral cellular or tendinous attachments have not been entirely divided, and the eye being again secured by the fore-finger or the elevator, the scissors are to be used, or the director is to be once more introduced, and the undivided part sought for and incised, when it will be found that the patient can no longer turn the eye inwards, and it is surprising how small a portion of attachment can do this, either on the under or upper part. In the earlier operations performed I was not aware of this circumstance, and in two cases in which a slight turn *inwards* re-appeared a fortnight after the operation, I attribute it to this portion of membrane, which in all probability adhered to the posterior end of the divided muscle. It may also effect this slight turn from its connexion either with the rectus superior above, or the inferior below, as the case may be. In three of the last cures performed the eyes would not turn outwards, although the sclerotic coat was rendered distinct to all present, for nearly three-eighths of an inch every way, which indeed ought always to be done to ensure success, and it was only by dividing the additional band of membrane or tendinous expansion I have alluded to in one case above, and in two below, that the operation was perfectly completed. The posterior cut end of the muscle is to be pushed backward by the director away from the other portion, and from the ball of the eye, so that it may unite indirectly to the posterior part of the globe and not to its side, and the edges of the incision in the conjunctival membrane are to be adjusted by the end of the same instrument. The eyes are then to be closed, by a small pad and a bandage, passed around the head ; the pad of the eye operated upon should be kept wet with cold water for the first twenty-four hours, and the eye closed for two or three days. These precautions are not absolutely necessary, but it is as well to observe them, and to apply a few leeches to the lower lid, if there should be any pain and swelling. The cut edges of the conjunctiva are prone to retract and swell and become elevated, whilst a small projection or growth takes place from the sub-conjunctival cellular membrane, which requires to be touched from time to time by the sulphate of copper, or the nitrate of silver, or even to be cut off with the curved scissors. The eye is bloodshot occasionally for some days. The operation is but a trivial one, and is not apparently very painful, although some persons complain of it much more than others, as is usual with all operations on the eye."

Such is the operation as it is now very generally performed.

Mr. Liston uses scissors, and holds the lower lid down with a spring tenaculum. This operation is rather longer, and certainly rougher than the common one.

There have been several proposals for simplifying the operation. Most of them refer to the specula. We shall notice one or two.

Mr. H. Attenburrow, of Nottingham, a very dexterous young surgeon, says :*

" The short-curved instrument I use for the upper eyelid, gently raising the tarsus with the fingers, and passing it underneath, so that the lid is completely supported, and may be raised at any time, if required, from the ball of the eye. The handle of the speculum lies on the patient's forehead. The longer instrument is applied in the same way to the inferior tarsus, and, from having its handle of a proper length (about seven or eight inches), the assistant's hand does not in the least interfere with the operator. With these specula I have been able to operate with ease on the most unruly children ; and another advantage is, that the patient can be allowed to rest by removing the instrument in any stage of the operation. In making the specula, care should be taken to obtain a proper curvature of the hooked portion which is intended to hold the eyelid, as, on its malformation, great pain will result on its application ; but any surgeon making the instrument had better apply it to his own eye, which will teach him the proper curvature. They are easily manufactured from copper wire, and the ends should be lacquered, which is effected by a spirit lamp."

In a note it is stated that—Coxeter of Grafton-street, Gower-street, has just invented a very simple and well-contrived apparatus for keeping down the lower lid without the aid of an assistant. It consists of a small speculum, by which the lower lid is held firmly, but gently downwards, and is retained in that position by means of a curved spring, fitted with a pad, which embraces the lower jaw.

*Mr. Clay, of Manchester,* has also invented a speculum. In general form and size it represents a teaspoon, having about one third of the extremity of the bowl removed by a semilunar cut ; on the external surface it is less convex than a spoon ; on the internal surface, that which is intended to be applied to the eye, it is concave and hollowed so as to fit the cornea and front of the globe, while the edges are thick to keep apart the eyelids. When placed in its position between the lids the inner angle of the eye is left uncovered, in consequence of the semilunar notch, and the surgeon is supposed to operate in the resulting space.

*Mr. Adams* proposes a knife of this sort :—it possesses all the properties

---

* Lancet, Sept. 12, 1840.

of a scalpel, namely, utility, effectiveness, and safety, in a smaller space than any other instrument with which I am acquainted that has been adopted for dividing the internal rectus muscle: the blade consists of two parts, a rounded portion, resembling the blunt part of a hernia knife, and a small flat sharp extremity, having a transverse cutting edge, which at one end terminates in a shoulder by uniting with a short sharp front edge, while its other extremity forms a point by meeting the back edge at right angles, so that we have the transverse effective part of the blade in immediate connection with the useful shoulder and point. The instrument described cannot be thrust into the eye by any sudden accidental resistance of the patient; and this safety is owing to the position of its straight cutting edge, which does not require any sharp part of the knife to be directed towards the globe of the eye during any moment of the operation: the muscle being slightly raised across the blunt hook, its fibres are divided transversely by the knife, its edges being directed towards the hook, but at right angles to it, so that the flat side of the blade is held towards the eye.*

But, after all, if we are to trust *Mr. D. O. Edwards,* these instruments are "all my eye." He, following the suggestions of *Mr. French,* does without them.

"The way in which I proceeded in all these was the following:—The patient was seated on a chair in a moderate light; an assistant standing behind covered the sound eye with one hand, and, with the other raised up the superior lid of the affected organ. The patient having been directed to turn the eye upwards and outwards, I snipped the conjunctiva at the under edge of the rectus, and passed a curved probe beneath the muscle: by depressing the handle of the probe the point was thrust forward, and appeared above the upper edge of the divided muscle, and by a cut of the scissors was enabled to emerge. The fibres were finally divided upon the probe.

The operation, thus abbreviated, consists of but three steps:—

First, the snipping of the conjunctiva.

Second, the introduction of the probe under the muscle.

Third, the cutting out of the probe.

The usual formidable notes of preparation are avoided. The probe which I employ consists of two stems placed parallel, at a distance sufficient for the point of the scissors to pass between. It is curved into a hook in the shape of a semicircle, of which the diameter is six lines. The pattern may be had at Savigny's or Fergusson's."†

*Mr. Duffin,* however, who informs us that he has operated *one hundred and seventy times,* and therefore ought to know something about it, laments the frequent failures which have happened, and thinks it not impossible that the operation may fall into disrepute. "When," says he, "it is imperfectly performed, a few fibres of the tendon, perhaps, or some apparently insignificant band of fibrous adhesion having escaped the scissors, the patient loses the power of turning the eye horizontally inwards, so as to bury the cornea in the nasal canthus to the same extent that he could do previously, and thus the inexperienced may be misled. If, in like manner, in his effort to accomplish this movement, it be found that the patient is still capable of directing the pupil either upwards or downwards, and only slightly inwards, we may rely upon it that the operation is incomplete, and that when the eye is left at rest, this modification of the original evil, will, in a minor degree, be found to persist. Now, the operator being satisfied, from the extent to which he may have laid bare the sclerotica, that he must have divided the whole of the tendon of the adductor, often erroneously concludes that this slight obliquity arises from sympathy, and that, in

* Med. Gazette, September 4, 1840. † Ibid.

the course of time, it will entirely disappear. But unless we can imagine that the unsevered parts will gradually relax, or become elongated, it is impossible that the eyeball can ever emancipate itself thoroughly from its confined position. I have lately examined several cases that were operated upon, when this mode of relieving strabismus was first practised in this country, and do not find that time has thus far effected any amelioration, nor do I think it at all probable that further improvement will ever take place; but the contrary.

When the operation is complete in every respect, the patient is wholly incapable of directing the pupil of the eye beyond the centre of the orbit, either in a horizontal or oblique line."*

When the eye can turn upwards and inwards, or downwards and inwards, it is probably because some tendinous or cellular attachments have been left undivided. They ought, therefore, to be perfectly severed.

---

### MONTROSE LUNATIC ASYLUM.

### 1. ABOLITION OF RESTRAINT.

In a Report of the Directors of this institution for the year ending on the 1st of June, 1840, we find some remarks by the able superintendent, Dr. Poole, on a subject which is now attracting some attention—the abolition of restraint. Dr. Poole observes:—

" As the subject of *restraint* has been recently brought forward in a variety of publications—and especially by Mr. Gardiner Hill of Lincoln, who contends for its total abolition in the treatment of the insane—I conceive it my duty to add a general opinion to these remarks, and it may be delivered in few words. My judgment, founded on many years' observation—and I shall make no pretensions to eminent humanity—is decidedly in favour of that gentleman's views, as either leading to or connected with the safest, easiest, most effectual, and, therefore, the best, system of practice for the cure of the deranged. I concur also with him in maintaining that, to render the plan adequate for all desirable purposes, 'several essential requisites must unite.' These are well stated by him:—' 1. A suitable building must be provided, in an airy and open situation, with ground sufficient for several court-yards, gardens, and pleasure-grounds, commanding (if possible) a pleasing and extensive prospect. 2. There must be a proper classification of the patients, more *especially by night*. 3. There must be also a sufficient number of strong, tall, and active attendants, whose remuneration must be such as to secure persons of good character, and steady principle, to undertake their arduous duties. And 4. The house-surgeon must exercise an unremitting control and inspection, in order that the plan may never, under any circumstances whatever, be deviated from in the slightest degree.'†

---

* Med. Gazette, September 11, 1840.

† " Mr. Hill, in a foot note, most truly says:—' Suicide under this system must be obviated by the constant attention of the house-surgeon to the proper classification of the patients *by night*. Those disposed to suicide should always be placed in an open dormitory under watch. *Nothing else can prevent suicide under any system whatever.*' For my own part, I do not recollect a single instance of its having taken place *in company;* and I entertain a belief that those who are disposed to perpetrate self-destruction would be among the foremost to arrest the hands of others who attempted it. The following paragraph, taken from the *Morning Chronicle* of 3d June last, though brief, affords a striking illustration of the difficulty experienced, under ordinary arrangements, in preventing such horrid deeds:—

My regret is, that the essentials now enumerated do not everywhere co-exist, and that Montrose cannot be held up as fully exemplifying both their combination and their highly probable results. In the absence of some of them, accordingly, and at variance with my own creed, I must tolerate the occasional imposition of hand-cuffs, to prevent greater evils than they inflict. Mr. H., I am persuaded, would not blame me, under circumstances, for departing from the *true faith*, inasmuch as, *e. g.*, I pinioned one man, because, having an ulcerated leg, which needed poultices and ointments, he repeatedly tore off and actually swallowed them; or that, with a latitudinarianism not deemed heretical in a *pure* physician, I had another tucked down to bed till the turbulence of delirium tremens yielded to a potent opiate; or even that, with only one female attendant for twenty of her sex, I permit the temporary confinement of a couple of arms which would both reduce their possessor to nudity and dispense merciless blows on all around. While I make these admissions in behalf of Mr. Hill's proposition, I confess the apparent force of an objection—for such is virtually the meaning of certain queries—lately put by an intelligent reporter. 'Even allowing,' says he, 'the practicability of the measure (total abolition of restraint), whether it be humane and desirable, is a question that appears to be very problematical. If the mind of a patient can be subdued by the power of intimidation, so as to paralyze his efforts in the hour of maniacal paroxysms, would his condition be more happy, or his welfare more promoted, by being under the influence of terror? Is it better to enslave the mind than enchain the body? May there not be greater benevolence and sympathy in subjecting the members

---

'*Suicide of a female lunatic in Bedlam.*—Last evening, at seven o'clock, an inquest was held before Mr. Payne, in the board-room of Bethlem Hospital, St. George's Fields, on the body of Jane White, aged forty-five, a lunatic, who committed suicide by hanging. Harriet Broady, one of the female keepers, said, deceased came first under her notice on Monday evening, when, on account of her uncleanly habits, she was removed from her regular ward to a cell on the basement storey. Witness put her in a strait-waistcoat, and she appeared very restless and melancholy. Witness had no difficulty in putting on the strait-waistcoat, and shortly afterwards she was placed in her bed Witness saw no more of her until between six and seven o'clock yesterday morning, when, on unlocking her cell door, she discovered her suspended by means of the strings of her strait-waistcoat from a piece of iron that fastened a pipe for carrying off the rain, a part of which pipe ran through the corner of the cell withinside of it. The house apothecary was instantly called, who cut down deceased, and pronounced her dead. By the Coroner: The waistcoat was put on properly, and secured in the usual manner, yet deceased contrived to get out of it. Deceased was also fastened down to her bed by a species of web-strapping, which she snapped asunder. By a Juror: It is usual to lock up refractory lunatics for ten or twelve hours together, and not to visit them during that period for fear of exciting still more their passions. Ann Thoms, another keeper, said, deceased had been under her care since February last, when she was first admitted from Stratford St. Mary's, Suffolk. She was always in a low desponding mood, and was esteemed the most inoffensive patient in all the hospital. Ann Powell, another nurse, said, the waistcoat was put on at her suggestion, after having reported the case to the matron. She was positive that the waistcoat was properly secured. The governor said that there had happened in the hospital only eight cases of suicide in the last twenty-five years, which was entirely owing to the extreme vigilance of the keepers, whom the patients were continually watching, to try and find an opportunity of destroying themselves. Verdict—Insanity.'"

of the body to salutary restraint than in the exercise of a moral discipline which will ever appear to human feeling burdensome and oppressive?'*

It will be observed that I used the phrase, 'apparent force.' In truth, there is nothing else presented by this extract. Mr. Hill might reply to it most cogently without abating one iota of that for which he contends, and simply because he would and must be among the very foremost to repudiate the assumed premises from which an inference opposed to his suggestion is deduced. To be brief, he does not argue for *terror*—neither does he mean to supersede bodily restraint by *a worse bondage of mind.* Moreover, it seems his deliberate conviction that the former, as usually practised, almost necessarily leads to, if not mainly operates by, the latter; and that, generally speaking, neither would be needed, if a moral discipline, not otherwise 'burdensome and oppressive' than is every check on violent passion, were more systematically exercised and more confidently trusted.

To conclude these observations—and the importance of the theme demands so many at least—while, in cases where intellect is totally suspended, as under the pressure of epilepsy, or in the rage of phrenitis, I should not hesitate a moment to guard against biting through the tongue by a wedge between the teeth, —or precipitation over a window by strapping down the limbs, the capability of reasoning, though in a very low degree, would be an argument with me for almost any species of mental influence as preferable to physical coercion. Imperfect as our classification may be reckoned, one immense benefit, I verily think, daily arises from it—if, so to speak, a negative can have a positive product—namely, that being, as it were, surrounded by an insuperable force—depending on the number of his associates, many of whom have the same tendencies to violence—each individual sees and feels, or, which is equal, imagines, that every outrage would be repressed instanter, and, consequently, worse than vain. Now, style this sort of influence 'the power of intimidation' or terror—suppose it to merit the name—and that it actually enslaves the mind—can I, ought I, to abandon it, at the sacrifice of peace, one of its obvious results, and have recourse, instead, to any mechanical devices whatever? My own answer is the stoutest—No. At the time when I entered on charge, the most dangerous patient in the house—a man deemed so ferocious and so filthy as to need perpetual hand-cuffing, with isolation from every living creature—was set at liberty by my direction. One of his first acts—he having been in the army—was to salute me *a la militaire:* he called me, shortly afterwards, an officer of his own regiment, and obeyed my commands accordingly: month by month did one or other human feeling acquire strength over his bad propensities: now he marches about for hours without molesting any one, causes no trouble to the Keeper, frequently converses very shrewdly, occasionally reads the Bible, and, though long ranked among the incurables, manifests faculties which my probably be cultivated into redemption from their grade. Another, equally unsafe at times, affects to be the guardian and protector of all who are feeble around him; and being, to a certain extent, entrusted with them, may often be seen officiating as a nurse—gently carrying one patient on his back to bed, washing and clothing a second, feeding a third, smiling and chattering to all. Yet, strange to say, as some may imagine, this is the very individual who, resenting the abstraction of an article confided to him, did not scruple a moment to punish the offender by an equally hearty blow. I might multiply instances in confirmation of the same principle; and it is incalculably cogent: Where judgment is not altoge-

---

* "Nineteenth Annual Report, &c. of the Dundee Royal Asylum, pp. 14, &c. My belief is, that, on coming to full mutual understanding of views, Mr. Hill and the Reporter, as well as myself, would be found essentially in concord."

ther destroyed, some portion of sympathy—the nucleus of moral character—may be reckoned on as co-existent with it; the treatment which a child receives from his parent is due to their possessor, and rarely fails, without odious allies, give them a triumph over the fiercest opponents."

2. *Benefits of the Penny Postage.*

" Among the hitherto unascertained though expected benefits of a diminished rate of postage charges, it is most agreeable and promising to all concerned, that an augmented interchange of reports and sentiments between the functionaries of Public Charitable Institutions has been experienced. No department of these is likely to be more advantaged by the facilities so afforded than lunatic asylums. Having already shared in the contributions thus promoted, I must express a desire that the managers will sanction similar returns on my part. They cannot do so without credit to their own charge, or aiding a cause appreciated by every true philanthropist."

---

### BETHLEM HOSPITAL.

### Report of the Commissioners for Inquiring into Hospitals.

We believe it must be admitted that great improvements do not always originate where the opportunities are greatest, nay, that into such favoured spots improvements sometimes penetrate with difficulty. Yet one would think that in the capital of this empire, where thought and action are practically more free than in any other country of the globe, our public institutions would be eager to catch and anxious to disseminate every spark, the very slightest, of advancing knowledge, and that nothing would be seen like a dogged adhesion to obsolete ideas and practices. The present condition of Bethlem Hospital would seem not to encourage these agreeable anticipations.

Mr. Martin has made, on the part of the Commissioners, a Report on this Institution, the conclusion of which we think it right to quote. We hope that the severe tone is not absolutely justified by the state of the case, yet we fear from what is mentioned in the body of the Report, that there is something like a foundation for it.

"The report is concluded by drawing comparisons between several parts of the system of management of the patients in Bethlem, with that adopted at other lunatic asylums, and especially those of Hanwell, St. Luke's, Lincoln, Glasgow, Nottingham, and Wakefield; and in all cases to the disadvantage of Bethlem. It is submitted that though to a casual visitor its perfect cleanliness, and the neatness of appearance and absence of all apparent restraint of the patients, might lead him to conclude that the management of lunatics has here attained perfection, yet there is still room for considerable improvement. Some of the defects in the present arrangement are, no doubt, to be attributed to the construction of the building, which appears to have been erected partly on the plan of the old hospital in Moorfields, with a view to the continuance of the coercive system there pursued, and is by no means well adapted to the present improved method of treatment. The aspect, it is said, is not good, the gallery windows facing to the north: the windows are too high from the floor, formerly more than six feet; the lower galleries ought not to be on a level with the earth, nor is there any necessity for stone floors in them. The centre of the building is obscured and made gloomy by a heavy useless portico, and the back is even more gloomy and repulsive than the front. The gloomy exterior of the hospital, and the heavy unsightly window-bars, savour strongly of the times of rigour and coercion; they are compared with great disadvantage with those in the lunatic asylums at Lincoln and at Nottingham, which are like common windows to external appearance, but are secured from being opened more than a certain distance. It is to be considered as much to be regretted, that the governors should have consented to the erection of the wings for criminal patients, by which the patients were deprived of the space now devoted to the use of objects who could not have been in the contemplation of the citizens when the existing lease was granted. The criminals now enjoy a larger quantity of ground in proportion to their numbers than the ordinary patients. The whole of the land held by the hospital appears no more than sufficient for the wants of the patients, but a very small portion only is devoted to their use. The kitchen gardens and the two lawns might be at once used or made fit for use as airing grounds, and the present airing grounds, which are mere bare cheerless inclosures, might be planted and rendered far more agreeable than at present.

Within the house the most striking feature is the want of occupation for the men, the greater number of whom may be seen sauntering about the galleries in listless and hopeless indifference. Some, indeed, play in the airing grounds, and a few are occasionally employed in the lawns and gardens, or in knitting; but the only general occupation is that of pumping at the crank and capstan. At Wakefield, Nottingham, Hanwell, Glasgow, and many other places, on the contrary, the occupations are of the most varied kinds, and the patients are even taught trades with which they were before unacquainted, and are employed as black-smiths, weavers, shoe-makers, carpenters, tailors, &c. The intellectual employment also of the patients at Bethlem appears capable of great improvement: only a very small portion of them occupy themselves in reading, and it was stated by the matron that the women, do not in fact enjoy the use of the library. Personal restraint also, though so much less than formerly, is still considerably greater and more frequently employed than in many other asylums.

Other suggestions for improvement relate to the too great caution which is evidently displayed before the patients, in everything around them being distinctly adapted to prevent them from harming themselves or others; the absence of any medical school or system of pupillage, by which medical science might be advanced, at the same time that it would (as it does in the general hospitals) afford a constant stimulus to the exertions of the medical officers; the propriety of having a resident physician, with suitable assistants and a remunerating salary, or of making an increase in the number of physicians, and, perhaps, an addition of one or two ordinary practitioners to those who devote themselves chiefly to the cure of insanity; the necessity of a greater portion of statistical information being afforded by the annual reports; the propriety of relaxing some of the restrictions by which certain classes are at present disqualified from admission, as those against persons with venereal disease or itch; and the importance of shortening the period between the consideration of the petition and the admission of the patient. The remaining strictures upon the hospital relate to the financial department both of itself and Bridewell, (with which it is urged its union is no longer reasonable), and they are made with a degree of severity which is entirely absent in the corresponding remarks on the other hospitals."*

The Governors, if they would stand well with the public, ought to look to this.

---

* Med. Gazette, April 17, 1840.

### On the Advantage of Studying Rare Cases.*

We could hardly have expected that, in this incredulous age, a champion could have appeared in behoof of the rare and the marvellous. Yet so it is, and he has thrown down the gauntlet to the matter of fact men pretty boldly. M. Lordat is the gentleman, and his eulogy of "the rare and strange" is contained in an Introductory Lecture to a Course of Physiology.

When, says M. Lordat, the old University of Paris was suppressed, and the republican government substituted the *Ecole de Santé* for the *Faculté de Medecine*, the director, besides his official duties, was appointed to explain the doctrines of Hippocrates, and the history of rare cases; that is to say, the history of the extraordinary phenomena which have been observed at various periods in some individuals, whether belonging to anatomy or physiology. It appears, however, that he did not give a single lecture on the latter subject; and in the catalogue of professors in the Parisian faculty of medicine, we no longer find one whose office it is to explain rare cases.

For our own parts, we think both the "Faculty" and the Professor lucky in not having such an office to boast of. We certainly should not like the job of explaining rare cases. But allons.

On examining, continues M. Lordat, the question more nearly, however, it seems to me that all parts of the history of rare cases are not equally neglected; cases belonging to anatomy are studied, but those appertaining to physiology are passed over. Monstrosities, anomalies in the distribution of the blood-vessels, club-feet, imperforations, and remarkable pathological phenomena belonging to surgery, are carefully collected. If they are not the subject of a special lectureship, they do not lack celebrity; they re-echo in the Academies; they are described at length and commented on in the journals; and they have been long mentioned in lectures on surgery and anatomy, with which they have more or less connection. But it is not so with the singularities observed in the exercise of the vital force of man, without material alteration. The facts formerly taught in the schools, and published under the names of *casus rariores, historia medica admiranda*, or *praxis miranda*, are now unknown; and those which are still presented to us by nature are unnoted, disdained, and rejected with derision.

Why are two kinds of facts, which are equally singular, treated so differently? We see, on reflection, that they are derived from the same source; that congenital deformities or monstrosities are the effects of the same cause which produces the most singular transitory phenomena, namely, the variations which happen in human *dynamism*. How is it possible to think so much of one kind, and so entirely to undervalue the other?

The immediate or proximate cause of this general disposition is to be found in the preference given by the majority to material knowledge over intellectual notions. The presence of a permanent fact which strikes the senses absorbs the whole attention: its origin and cause are forgotten, while the observer is occupied in classing it, or employing it for some mechanical purpose. But a vital, fugitive, and extraordinary phenomenon is often noted by the skilful alone; it vanishes before a sufficient number of witnesses have been able to satisfy themselves of its reality, and make it sufficiently notorious. Since nothing visible remains of it, we can only investigate its relations, affinities, and causes; an intellectual labour which is very difficult, and is no longer the mode of the day.

The disregard of this study proceeds from the prevailing error of neglecting the theory of physic, and believing that the causes which we have seen are suffi-

---

* Med. Gaz. Sept. 18, 1840. From the Gazette Medicale.

cient to form a complete nosology. The majority of physicians have forgotten the necessary truth, that the number of things which the most busy practitioner has seen is infinitely smaller than the number of those which he has not seen. From this forgetfulness proceed the disuse of many doctrinal principles of a high order, the ignorance of many vital facts necessary to be known, contempt for the past in medicine, profound indifference for anthropologic erudition, a great desire to appear well-informed in rare cases of the anatomical kind, and an extreme wish to show oneself an *esprit fort* (incredulous) with respect to these singular phenomena in the domain of physiology, which we have not witnessed ourselves. Nay more, we now actually see men, otherwise estimable, who after applying the test of their senses, quite at their ease, to physiological phenomena differing from those with which they are familiar, seriously repeat what Fontanelle uttered as an epigram: "*Je l'ai vu, et je ne le crois pas.*"

We believe we can account for what puzzles M. Lordat. The physiological, and, indeed, the medical world has seen so many blunders and so many lies in reference to the extraordinary that it has grown sceptical. And it could not well grow wise without disbelieving half that is told. What then are we to do? Open our ears again to the physiological Pintos, who exist even at present and would bask in the sunshine of public credulity did it shine upon them? No, no M. Lordat, the time for all that is gone by, the spiritualisms and mysteries of transcendental philosophy will never take deep root with the children of this generation. A few hot visionaries or weak neophytes may be caught, but the majority like something substantial, appreciable, and satisfactory. They recollect homœopathy, animal magnetism, and those other collections of *rare cases*, which have proved so veritable and so instructive.

---

### MERCER'S HOSPITAL, DUBLIN.

### New Instrument for Tapping.

Dr. Lendrick objects to the old trocar and canula, perhaps rather unkindly. Our contemporary* who states this, states also that the Doctor shewed the pupils a new instrument, made under his direction by Mr. Milliken, of Grafton-street. He stated that he was not aware of a similar plan having been resorted to. The instrument is the "exploring needle," on a large scale, as to breadth, being about three inches long, two lines and a half broad, and one line and a quarter deep. It has an oblique handle, like that of a gorget. There is a groove on the surface, and the extremity is lancet-shaped, with only two edges, extending not quite to the shoulders of the blade. The integuments may or may not be divided with a lancet, according to the wish of the operator. The instrument is introduced with great ease, and the penetration of the sac is at once known by the gush of fluid along the groove: the operator, who holds in his hand a catheter (No. 4 or 5,) nearly straight, and either of silver or elastic gum, immediately passes it along the groove, and its penetration of the sac is simultaneous with the withdrawal of the sharp instrument. The fluid is now allowed to flow through the catheter, pressure with a swathe being applied as the abdomen becomes flaccid. The whole operation may be performed without varying the position of the patient from the recumbent posture in bed. The wound is somewhat valvular. The operator ought to hold the trocar in one hand, and the catheter in the other. The introduction of the second is to be accompanied by

---

* Med. Gaz. Aug. 14, 1840.

the withdrawal of the first. Thus the aperture of the sac is at once hit off. One precaution is necessary to be attended to, if a gum catheter be used. After the stylet is withdrawn, the tube is liable to be compressed by the edge of the swathe, and the exit of the fluid impeded. The difficulty is overcome simply by removing the edge of the swathe from the orifice. It is preferable that the catheter should be furnished with two apertures.

We fancy the trocar and canula will have the best of it.

---

### DREADNOUGHT HOSPITAL SHIP.

### Condition of an Elbow nearly Three Years after Excision of the Joint.

Facts like the following, which shew the *ultimate* results of remedies are valuable, and, unfortunately, no less valuable than rare.

A man had the upper extremities of the ulna and the radius, and the lower extremity of the radius excised by Mr. Busk, the able surgeon of the Dreadnought, in November, 1837. About a year after the operation, the patient went to sea, and did his duty on board ship.

" He presented himself to me on the 6th of this month, when I had an opportunity of fully examining the condition of the limb. Before removing his clothes it required close observation to detect any difference between the limbs, and he stated that no one on board the vessel of which he had been mate for nearly twelve months was aware that he had any thing the matter with his arm. On baring the arms, however, there was considerable difference in their size, although the muscles of the right had much increased since I had seen him last. The affected arm was altogether smaller than the other, but was not otherwise at all deformed. The shape of the elbow was so little altered that any one not aware of the fact would hardly have been able to convince himself that the articulation had been removed. The arm could not be flexed to more than a right angle, but could be perfectly extended. There was no lateral motion. The motions of supination and pronation of the hand were perfect. He had no pain, and found only that the right arm was not as strong as the left. He had become very stout, and appeared to enjoy the most robust health."*

---

### HOTEL DIEU.

### Barbarous Operation for Fistula, by M. Roux.

Dr. J. C. Hall, in a sensible paper on Fistula Ani, gives this account of M. Roux's operation for fistula. It is worthy the times of the Grand Monarque.

He, (M. Roux, *not* the Grand Monarque,) introduces a long piece of box-wood into the rectum, having its concavity towards the fistula. A silver director is then introduced along the fistulous track, and its end made to come in contact with the wooden gorget in the bowel. A long, strong, narrow sharp-pointed knife is then introduced along it, till it comes in contact with the piece of box-wood. The director is then withdrawn, and by keeping the point of the knife fixed upon the gorget, and withdrawing both together, all the parts between the fistula and the rectum are divided. This part of the operation completed, the

---

* Med. Gaz. July 24, 1840.

bistoury is exchanged for a scalpel, and all the hardened base is carefully dissected out. A thick long probe is then procured, having a button at one end; this is covered with charpie, smeared over with some yellow-looking ointment, and the wound crammed full of it to the bottom.*

### GLASGOW ROYAL INFIRMARY AND LOCK HOSPITAL.

### Dangers from the Exhibition of the Iodide of Potassium.

Dr. Lawrie, physician to the above Institutions, relates several cases with the view of pointing out the dangerous consequences that may follow the use of the iodide of potassium. The cases we need not quote—the conclusions we may.

"It would appear," says he, "that the hydriodate of potash and iodide of starch are dangerous and uncertain remedies. I am, in my own mind, quite satisfied that they were the causes of death in cases 3d and 5th. Their uncertainty, in a remedial point of view, is even more to be lamented than their danger. If they were unsafe in large doses, and safe in small, or if the disease for which they are exhibited, or the constitution of our patient, had any definite influence on their poisonous effect, they might be used with comparative impunity. As yet, however, I know of no criterion by which we can judge beforehand of their probable effect; that the quantity exhibited is no guide, I am very certain. Case 3d had only taken one grain of the solid iodine twice daily for five days, when he died, while many of Dr. A. Buchanan's patients take *seventy-two* grains of iodine daily, in the same form, without any bad effects, for days and weeks together. Case 5th took five grains twice daily for a week, when she died; while Wm. M'Symont, æt. 50, Ward 7, with gangrene of the penis, took ʒj. daily for eleven days, with apparently great benefit to his disease, and not one of its injurious effects. I have given it in double this dose, and known half an ounce given daily with impunity. I very much regret this danger and uncertainty. I consider hydriodate of potash as by far the best of our recent remedies, and have prescribed it more frequently than any other medicine. In future, however, I shall be more cautious in its employment. Every patient for whom it is prescribed should be frequently seen by his medical attendant, and should be warned to omit it whenever any of its constitutional effects appear. Case 5th teaches us to omit it when the papular eruption is profuse, and not to resume it. Swelling of the neck, hoarseness, and dyspnœa (cases 3d and 5th,) are most dangerous symptoms. For them I believe there is no remedy but tracheotomy, early performed, and on it I would place great reliance. Case 5th teaches us that delay is fatal; and I would not in any future case allow the state of the bronchial mucous membrane to deter me from its performance.

If the above observations are correct, it would further appear that these iodides exert their poisonous influence on the mucous membranes of the air passages, not as direct irritants, but indirectly through the circulation, in the form of acute inflammation. I have never seen them act as irritants to the gastro-intestinal mucous membranes, nor have I ever seen them produce emaciation, atrophy of the mammæ and testicle, hectic, and those other symptoms described by various writers under the name Iodinia."†

We prescribe the iodide a good deal, and we cannot say that we ever saw much mischief from it. Slight salivation, and some irritation of the gastro-in-

* Med. Gaz. July 3, 1840. † Ibid.

testinal mucous membrane we have seen—nothing more. But then we give it with caution—in moderate doses—*not* when there are, or have just been pyrexia or inflammatory symptoms—and we stop it when it disagrees.

---

### PENNSYLVANIA HOSPITAL.

### Statistical Account of the Cases of Amputation performed at the Pennsylvania Hospital, from January 1, 1838, to January 1, 1840.

The application of statistics to the great operations, is likely to be very serviceable. With what horror have we regarded lithotomy? It has been looked on as the most painful and fatal of operations. Yet it is not near so fatal as amputation, nor, perhaps, as most of the great operations.

Dr. Norris, one of the surgeons to the Pennsylvania Hospital, has published an account of the result of amputations performed in the institution. He alludes to a similar account published by Mr. Phillips, in the Medical Gazette. The number of cases collected by him is 640, embracing all cases, acute, chronic, and the results of violence which occurred in the practice of the persons by whom the returns were furnished within the period of four years. "Of these cases, 490 are reported cured, and 150 died, either in consequence of the operation or the progress of the disease, to rescue the patient from which, recourse was had to the operation."

Dr. Norris observes:—

"The necessity of extending any observations that may be made through a term of years, is strikingly shown by an inspection of the tables which I have made; in some years the mortality after these operations being very small, while in others, though a similar class of cases have come under notice, and been subjected in every way to similar influences and treatment, the mortality has been large. From the first of January, 1830, to the first of January, 1832, but one death took place out of eleven amputations made during that period, while from the first of January, 1832, to the first of January, 1834, one-half of those amputated died (seven out of fourteen), and in the next succeeding two years the mortality became still greater, eight out of fifteen terminating fatally. From 1836 to 1838, the mortality then strikingly decreased, the loss being only one-third (five out of fifteen) and by the accompanying table it will be seen that, from the first of January, 1838, to the first of January, 1840, there has been but a single death out of twenty-four amputations, seventeen successive operations having had a favourable termination. To assert that death after amputation is rare with us, would be warranted by the experience of the past two years, though undoubtedly it would be as far from giving a true idea of the danger of the operation, or of our average success, as to aver our ordinary results to be such as were had between the years 1834 and 1836."

For reasons which seem satisfactory, but which we need not state, Dr. Norris concludes that the success at the Pennsylvania Hospital represents sufficiently that in the other large hospitals of America. All those amputations performed within twenty-four hours after admission, are included under the head of immediate, the patient in such cases having been brought to the house soon after the receipt of his injury. With one exception, the common circular operation was performed, and the stumps were all dressed so as to procure union by the first intention. The ordinary mode of dressing, is first to bring the flaps together by means of three or four long strips of adhesive plaster, and after covering the lips of the wound with lint spread with cerate, to apply a small cushion of charpie over the extremity of the stump, and to secure the whole with a bandage moderately tight. The first dressing was generally made on the third or fourth day, and repeated daily afterwards till cicatrization was complete.

Dr. Norris furnishes a table of the amputations performed in the hospital from January 1, 1838, to January 1, 1840. That table we need not introduce, but content ourselves with citing the results of the amputations in question, added to those of the eight previous years.

Of eighty amputations on 79 patients, performed during a term of ten years at the Pennsylvania Hospital, thirty-five were primary, of which twenty-four .were cured and eleven died, four of the deaths occurring within the twenty-four hours immediately following it.

Twenty were secondary, of which thirteen were cured and seven died.

Twenty-five* were for the cure of chronic affections, of which twenty were cured and four died.

Thirty-two of the amputations were of the upper extremity, of which twenty-seven were cured and five died.

Forty-seven were of the lower extremity, of which thirty-one were cured and sixteen died.

Seven were amputations at the joints, of which four were cured and three died.

| | | | |
|---|---|---|---|
| 13 of the 79 operated on, | were under 20 years of age, | of whom 12 were cured, and | 1 died. |
| 26 | were between 20 and 30, | of whom 19 were cured, and | 7 died. |
| 22 | were between 30 and 40, | of whom 15 were cured, and | 7 died. |
| 16 | were between 40 and 50, | of whom 9 were cured, and | 7 died. |
| 2 | were upwards of 50, | of whom 2 were cured. | |
| 79 | | 57 | 22 |

The following are Dr. Norris's conclusions :—

1. That amputation† with us is to be regarded as an operation attended with much danger to the life of the individual, the mortality after it being one in $3\frac{7}{11}$.

2. That the chances of success after it are much greater in persons who have been for some time suffering from chronic diseases, than in those who have it done while in robust health, the mortality in the former class of cases being one in $6\frac{1}{4}$, while in the latter it is one in $3\frac{2}{11}$.

3. That immediate amputations after injuries are less fatal than secondary operations, the mortality after the former being one in $3\frac{2}{11}$, while in the latter it is one in $2\frac{6}{7}$,

4. That amputation of the lower extremity is much more fatal than that of the superior member, the mortality after the former being one in $2\frac{15}{16}$, while in the last mentioned class of cases it is only one in $6\frac{2}{5}$, and

5. That the danger increases with the age of the individual operated on.—*Amer. Journ. of Med. Sciences, May,* 1840.

---

* One double.

† The *great* amputations only, it will be recollected, are alluded to. No death has followed any of the amputations of fingers, or toes, which have been made in the hospital during the ten years past.

REPORT OF SURGICAL CASES TREATED DURING THE MONTHS OF JULY, AUGUST, SEPTEMBER, AND OCTOBER, 1839. By G. W. NORRIS, M.D. one of the Surgeons to the Hospital.*

FROM this Report we shall extract the more interesting cases.

### I. UN-UNITED FRACTURE.

1. *Un-united Fracture of the Bones of the Fore-Arm of four weeks standing. Treatment by perfect rest and pressure—Cure.*—Henry Baldwin, a healthy boy, ætat. 12, was admitted July 31st. His mother stated that his accident had been produced by a fall four weeks previously, and that his arm had been attended to by a physician in the country. Upon examination the fracture was found to be near the middle of the fore-arm; neither of the bones had united, considerable deformity existed, from bending of the bones outwards, and the integument was slightly ulcerated over the projecting ulna. The fore-arm was secured to a splint extending from the elbow beyond the fingers, and as much pressure was made over the projecting fragments, as the patient was able to bear. At first the bandage was removed, and the pressure re-applied daily, and, after a short time, every second or third day. By the 20th of August, perfect union had taken place, and the limb had become much straighter. Pressure and the splint, however, were continued till the 4th of September, and on the 28th he was discharged perfectly well, and with a very slight deformity at the part.

2. *Un-united Fracture of the Femur of seven months standing—Treatment by rest and compression.*—Michael Ward, ætat. 50, and enjoying good general health, was admitted September 14th, with an un-united fracture of the femur. He states that he received his accident in the middle of last February, by the fall of a bank of earth upon the limb. His left femur was broken obliquely in its lower third, together with both bones of the leg of the same side a short distance below the knee. After a treatment of seven or eight weeks, with long splints (probably Desault's) he was suffered to get up and move about, the union of the bones of the leg being firm, though somewhat deformed, while the thigh was much deformed and un-united. In August he set out from Ohio for Philadelphia, and performed one-half the distance on foot, with the aid of crutches. On admission, the left limb was found to be two and a half inches shorter than that of the opposite side. The lower part of the thigh was enlarged, owing to a great mass of callus which had been thrown out around the seat of fracture, and was much bent outwards, the lower fragment being drawn to the outer side of the limb. When placed in the erect position, he was unable to bear any weight upon the limb, and upon attempting to do so the motion of the lower upon the upper fragment, was very evident both to the patient and observer. Strong extension had no effect in bringing down the limb to its natural length, and all that was proposed by the treatment was to produce union of the fragments, and, if possible, a diminution of the deformity, by rest and pressure, without attempting to remedy the shortening. For this purpose a roller was applied from the toes upwards, after which strong pressure was made over the projecting portions of bone by means of a bandage secured to a well padded long splint placed upon the inner side of the limb. This apparatus was continued until October 14th, the bandage being tightened every

---

* Amer. Journ. of Med. Sciences.

few days. At this date the limb was found to be free from swelling, and the deformity evidently less. Union also appeared to be firmer. The apparatus was re-applied, and in order to keep up as much pressure as the patient was able to bear, a tourniquet was applied immediately over the point of fracture. On the 31st of October the apparatus was removed for the purpose of accurate examination, and it was found that the limb was lengthened half an inch since the commencement of the treatment; that the deformity was less, apparently from absorption of a portion of the callus which had been deposited on the outer part of the limb, and that no motion could be discovered at the point of fracture, or any grating sensation perceived when the weight of the body was borne upon it.

Dr. Norris observes that, in the first case, the non-union was probably the result of the want of proper support and pressure—in the second case, of the same circumstances, in addition to the existence of a fracture of the leg. For, where more than one fracture exists in the same person, firm union may take place in one some time before it occurs in the other. Pressure and rest are best adapted to cases of no long duration, and to young persons. Dr. Norris prefers the tourniquet to other means.

### II. Rupture of the Ligament of the Patella.

James Hughes, ætat. 30, was admitted on the evening of July 13th, for an injury of the knee received a short time previously by a fall from a height of eight feet into a well. He states that his limb was strongly flexed at the time of the fall, and that he struck the ground with his knee. Though suffering great pain he was able to seize a rope which was lowered to him, and allow himself to be drawn up to the surface, but on attempting to rise upon his feet he found that he was unable to support himself on his left limb. He was immediately removed to the hospital, where upon examination by the resident surgeon, a hollow was observed over the knee which on first sight was supposed to result from fracture of the patella, and separation of the fragments, but which on close examination was seen to proceed from a rupture of the ligament of the patella and a consequent displacement of that bone upwards. On the next day, although there was swelling, Dr. Norris found a deep depression existing immediately above the tubercle of the tibia, and the patella drawn up to the extent of an inch and a half. This bone admitted of a much greater degree of lateral motion than that of the sound side, could be distinctly traced, and was found to be uninjured. The treatment consisted in extending the limb upon a splint, and applying a roller from the foot upwards, which was passed around the knee in such a manner as to draw down the patella towards the head of the tibia. The limb was then placed on an inclined plane, and cold applications applied over the knee. Nothing of great moment happened, and, on the 27th, the patient spontaneously left the house. The patella occupied its natural position, and a considerable hardness could be felt in the space between its lower edge and the tubercle of the tibia.

### III. Wound of the Palm of the Hand—Secondary Hæmorrhage—Ligature of the Radial Artery—Return of Hæmorrhage—Cure by Pressure.

H. L. farmer, admitted July 22. He states that four weeks since, he received a cut with the end of a scythe in the fleshy part of the palm about an inch below the end of the radius. The injury was followed by considerable hæmorrhage, which was arrested by means of pressure on the part. The wound from the time of the accident gave no trouble, though it did not cicatrize, until eight days since, when considerable hæmorrhage took place from it. This was repeated at different times during the day, and the following morning he came to the city and applied to a surgeon, who in order to arrest it, secured the radial artery just above the wrist. After this he returned home and continued to do well until the night before his admission into the hospital, when the wound again bled profusely, and was only arrested by making very considerable pressure upon the wrist as well as over the wound. After admission, the pressure which had been hurriedly and irregularly applied, and which caused great pain, was removed, and the wound which was not above half an inch in length was sponged off, but no bleeding took place. The ligature upon the radial artery, had not yet separated; the incision made in order to secure it had not united, and its edges were much swollen and inflamed, as was also the back of the hand. Moderate pressure, by means of graduated compresses and a bandage, was made over the wound and lower part of the ulnar artery, and the hand and fore-arm were secured to a splint and elevated, and cold applied. Tinct. opii, gtts. xl. given, and absolute rest in bed enjoined.

On the 1st of August, the ligature separated from the radial artery; and, on the 7th, the wound was cicatrized, and the patient discharged.

Dr. Norris remarks that, in wounds of the arteries of the wrist or palm, if the wound be large and the ends of the vessel accessible, everybody admits that both ends should be tied. But, where those ends cannot well be got at, the practice is to secure the vessel above. "Where the case is recent, this method is mostly successful, but when any length of time has elapsed since the injury, particularly in wounds of the palm, where the anastomoses between the vessels are free, it very frequently fails, the hæmorrhage after a time returning by the inferior end of the vessel. The employment of graduated compression upon the vessels of the fore-arm as well as over the wound, was in the above case fully successful in preventing any return of the hæmorrhage, and is applicable to most similar instances, but in all cases in which this is trusted to, it should be recollected that to ensure success, the pressure must be well and evenly applied, and a state of perfect rest secured to the limbs by means of a splint: elevating the limb, too, is a most important matter where this method is pursued."

### IV. Difficulty of Diagnosis of Injuries about the Hip-joint.

Dr. Norris relates several cases in which mistakes in diagnosis were committed. In one, disease of the hip-joint was mistaken for luxation—in a second, luxation for fracture—in a third, fracture was at first overlooked—in a fourth and fifth, the same error was committed. The cases themselves we need not detail—it will be enough to extract a few cautionary observations of Dr. Norris.

*a.* "The true nature of the injury in these cases is often more evident some hours or days after the receipt of the accident, than immediately after its occurrence, and I am inclined to think that the necessity of close *secondary* examinations, in all instances in which there is room for a doubt as to the nature of the injury, are not sufficiently insisted on. Had careful examinations of the first two of the above cases been made the day following the receipt of the injuries, it is hardly possible that their true natures would have been mistaken, and the symptoms of fracture in the third case were only detected after a lapse of some days, though this injury was at first suspected and the patient attentively examined. It is, however, more particularly where fractures may be suspected that these repeated examinations are demanded."

*b.* "The exact length of the limb on entrance, with the natural position of the foot and absence of any deformity, or crepitus, led to the supposition, in both of the above cases, despite the great pain suffered, that simple contusions only of the part existed, and this idea was confirmed at the commencement of the attacks of mania a potu, upon seeing the men up and moving about the room; but after recovery from their attacks, the eversion of the foot led to an immediate examination, when the shortening was found to exist, which, in connexion with the symptom just mentioned, could only be caused by fracture of the neck of the bone. In both cases the fragments must have been interlocked in such a way as to have prevented any shortening or motion, and so remained till the delirium occurred, when the violent efforts made to use the limb, unlocked the parts, and permitted the lower fragment to be drawn upwards.

Previously to witnessing these cases we had believed it impossible for a patient with fracture of the neck of the femur to walk upon the limb: but upon examining the records of our science on this point, I find that similar instances are noted."

And he quotes Sabatier, Dessault, Dr. M'Tyer, Mr. Syme, and M. Malle.

### V. Cancer of the Breast in a Male.

A. C. æt. 61, admitted July 18. He states that fifteen years since, while in the act of loading a cart with wood, he was struck in the breast by a heavy log. Soon after this, a small lump, not much larger than a pea, was observed by him at the part, which was hard and apparently loose beneath the skin, and not painful. For some years this never increased; but during the last eight or ten years, when the part was at all pressed upon, or rubbed by his suspender, it would inflame —at times so much so as to induce him to apply a soft poultice to it, which always speedily removed it. During this period, too, it increased very slowly in size. At present the tumor is about the size of a small apple—is extremely hard, and in no way adherent to the parts beneath—is not painful when handled, and has a small and very superficial ulceration on its outer part. He states that the tumor attained the size which it now has during the last summer, and that the slight abrasion of its surface occurred three weeks since. The skin covering the tumor is in no way discoloured, and but a single lymphatic gland, immediately above it, can be discovered to be enlarged. General health good. On the 20th, the tumor was removed. On cutting into it, it was found to consist of a whitish shining mass, almost of the consistence of cartilage, which gave a crying sound when divided by the scalpel, and showed numerous white striated bands running from its centre into the surrounding cellular structure.

The wound was healed on the 18th of September.

### VI. Compound Fracture of the Cranium.

Dr. Norris relates two cases of this sort of injury—one that interests and always must interest surgeons, from the questions which its treatment involves.

*Case* 1.—J. F. æt. 16, admitted July 16, a. m. He had got entangled in a steam-engine, which had gradually forced his head up against a projecting bolt. This bolt, which was square at is projecting extremity, and measured three quarters of an inch on either side, entered the fore part of the head to the depth of an inch and a half. From this situation the workmen attempted to extricate him, but finding this impossible, separated the bolt, together with a heavy piece of iron to which it was attached, from the machinery, leaving it firmly fixed in the skull. Attempts were then again made, by the foreman of the shop, to remove the bolt, but without success, and a medical gentleman in the neighbourhood was sent for. The opening in the forehead was of such small extent that it was found necessary to enlarge the wound in the soft parts slightly at its corners, and afterwards to turn the bolt until its angles corresponded to the corners of the wound before it could be withdrawn. Two or three small portions of bone, which had been driven in upon the brain, were removed after the withdrawal of the bolt. At the visit soon after his entrance into the hospital, the seat of injury was found to be at the anterior part of the frontal bone an inch above the orbitar ridge, and a little to the left of the middle line. At this point a portion of brain, judged to be nearly equal in size to a large walnut, was protruding and much lacerated. The patient was sensible and complained of no pain; pupils natural; pulse slow and feeble; is unable to raise the right arm from the bed, though he is able to close his fingers feebly, and has perfect sensation in it; no paralysis of any other part of the body. A pledget of lint was applied to the wound, and ice-water to the forehead. The treatment was antiphlogistic, and we need not particularly specify it nor the symptoms in detail.

On the 3rd, the protruding portion of bone had in a great measure disappeared. The mind was little, if at all affected, nor was there fever to any amount. On the 5th, the wound had begun to suppurate. On the 9th, he could raise the right fore-arm from the bed. In a day or two, he began to complain of pain over the eyes, which he did, at intervals, subsequently. On the 19th, the paralysis had entirely left the arm and he was able to raise it completely from the bed. From this date up to the 18th of August, the patient continuing to do well, the wound presenting a healthy appearance, and cicatrization progressing slowly. On the evening of the day mentioned he was seized with a chill followed by fever, pain in the head and diarrhœa. But on that day (the 19th,) there was pain in the head and pyrexia. He was bled. On the 21st the discharge was in a much less quantity, and from this time up to the 30th, the day of his death, continued to be very trifling. A day or two previous to his death his memory was slightly affected, and he was observed to sleep sounder and more than he had previously done; no paralysis. Early on the morning of the 29th he was much as he had been on the previous day, but at mid-day was found to be in a state of profound stupor with the pupil of the right eye strongly contracted, while that of the left side was dilated. In this state he continued till early in the morning of the 30th, when he died.

*Autopsy* nine hours after death. The wound in the head was one and a half inches above the orbitar ridge. A small piece of bone was driven in and pressing upon the brain. The dura mater around the wound was thickened and strongly adherent to the brain. The anterior lobe of the left hemisphere of the brain was of a bluish-yellow colour, softened and presenting evident fluctuation. The vessels of the pia mater were much injected; an abscess was found immediately beneath the dura mater at the point at which it was adherent to the brain, occupying the whole anterior lobe and filled with thick yellow pus—thought to amount to ℥v. or ℥vi. The walls of the abscess were lined by a thick false membrane of a grayish colour. The cerebral matter around it was of a light yellowish colour and softened. A portion of the internal table of the bone, about an inch and a quarter in length, was found driven in beneath the sound bone at the upper part of the wound, and at the side of it, a small fragment was found firmly attached to it. The opposite hemisphere of the brain presented the natural appearances; the ventricles were much distended with serum; the edges of the fractured bone were rounded off and perfectly smooth. No further examination was admitted by his friends.

Dr. Norris observes, that the above case is remarkable from the large size which the abscess had attained, as well as from the length of time the patient survived after the accident, free from all unpleasant symptoms. The knowledge that the loose portions of bone had been removed immediately after the accident and the entire freedom from all symptoms of compression of the brain, were sufficient to prevent a resort to any operative procedure.

But we would observe, that from the circumstance of bone being driven in and pressing on the brain, this case is, pro tanto, evidence against the plan of letting compound fracture of the cranium, with depression, remain without operation. The principle of the latter is, that, so much mischief being done to the brain, a little more will probably do little harm, and, if it removes all sources of irritation, will probably do good.

*Case* 2.—W. M. aged 34, ostler, was admitted December 3, 1832, for compound fracture of the cranium with depression of the bone. His injury had been produced two hours before admission by a blow over the right side of the head with an axe. The wound in the scalp was four inches in length, and a fracture with some depression of the bone existed. The physician who saw him previous to admission, stated that a small portion of brain had escaped from the wound. He had lost a large quantity of blood, and had no symptom of compression of the brain, or uneasiness of any kind about his head. The lips of the wound were brought together with adhesive plaster, and cold was applied to his head.

Under a very rigid antiphlogistic treatment he did well till the 13th, when he was observed to sleep more than usual, and when aroused said that his head felt heavy and painful. No inflammation existed round the wound, which had partially united. Thin pus was discharged from it, and it had for some days previously been dressed with a poultice. Cups were applied to his temples and back of the neck, and he was purged with salts and put upon the use of nitrous powders. He stated that some relief had been afforded him by the cupping, though on the 14th he complained of his head being still heavy, and slept a great deal and heavily. His pulse had become slower (60); skin dry and warm. The 15th, he was more stupid, and complained of pain across his forehead. *Head shaved and blistered—saline solution.* By the 20th the blister was healed, and the pain in the head was gone.

On the 22nd, it was discovered that he had not complete power over his left arm. Yet he continued to improve from this date till the 8th of January, 1833, when he complained of pain in his forehead, for which he was cupped and purged with relief. By this time he had completely regained the use of the left side.

On the 9th he had a return of the pain, and he again lost the use of the left arm. The pain was again removed by cupping and purging, and two days after he had partially regained the use of the member.

On the 16th a piece of the outer table of the bone, an inch in length, was found to be loose within the opening, and was removed.

On the 23d he was suffered to walk about the ward, and had his diet slightly increased; the wound had healed with the exception of a fistulous orifice leading to the seat of fracture. His mind was perfect; he suffered no pain, but had only an imperfect use of the arm of the left side.

He continued, as at the last report, until the 26th of March, when he was seized suddenly with a convulsion; for this, cupping and cold to the head were again resorted to with relief. The fistulous opening still remained, from which there was daily a discharge of pus.

In consequence of this attack, it was judged proper to cut down to the bone and ascertain whether or not any fragment pressed upon the brain; this was accordingly done, but no projecting portion was discovered. No material change took place from this time till the 2nd of April, when he complained of headache, which was in a short time followed by vomiting, insensibility and death on the same day.

Upon examination, eighteen hours after death, it was found that the internal table of the fractured portion of bone had been driven under the sound bone, and had become firmly adherent to it; and that an abscess occupying the greatest portion of the anterior and middle lobes of the brain, existed on the right side of the head, communicating with the external wound and lateral ventricle.

As Dr. Norris observes, this case shews the necessity of caution in prognosis and in treatment after injuries of the head.

---

### MASSACHUSETTS GENERAL HOSPITAL.

### STATISTICS OF THE AMPUTATIONS OF LARGE LIMBS.*

The report from which we draw the following particulars is from the pen of Dr. Hayward, one of the surgeons to the hospital.

Dr. Hayward well observes, that the fatality of amputations has taken people by surprise. More than one-half of the amputated die in the Parisian hospitals; and of fifty-five so operated on in the Pennsylvania Hospital, twenty-one died. Amputation is, therefore, a serious evil, and should not be lightly had recourse to.

The results at the Massachusetts Hospital were somewhat more favourable than those at the Paris, and Pennsylvania Hospitals above referred to. In a large proportion of the following cases, the amputation was done by the circular incision; the flap operation was adopted occasionally, whenever there was reason to believe that a better stump could be made by it than by the other method. The dressings were always of a light and simple kind; consisting of two or three strips of adhesive plaster and a small compress and roller; and yet there are some surgeons of the present day, who would perhaps regard these as more cumbersome than was necessary.

If the bleeding was slight, the dressings were applied before the patient left the operating room; but if there was any thing more than an oozing from the veins, it was deferred till a few hours after.

Secondary hæmorrhage was not frequent, though it sometimes occurred; pressure was generally sufficient to arrest it, but occasionally it was found necessary to open the stump, and tie one or more vessels. In one case where hæmorrhage occurred twelve days after the operation, from a diseased state of the posterior tibial artery, the femoral artery was tied. No one who had secondary hæmorrhage died, and though it sometimes debilitated the patient, in no case was there any permanently injurious effect from it.

In all the cases it was attempted to heal the wound by the first intention, and in a few instances it was completely successful, but in by far the greater number it was only partially so.

It has not been the usual practice at the Massachusetts Hospital to administer an opiate before an operation, though in a few instances it has been done. In one case where amputation was performed on a patient with delirium tremens, twelve grains of opium were given shortly before the operation; he became drowsy soon after and recovered.

Dr. Hayward subjoins a table of the amputations performed in the hospital. This is too long for insertion, and we may content ourselves with presenting the summary.

---

* Amer. Med. Journ. May, 1840.

There were seventy operations on sixty-seven patients; three patients having two limbs removed. In one of these three cases, one operation was above and the other below the knee, and in the other two, both operations were below; the first patient died, and the other two did well.

Of the whole number operated on, fifteen died and the remainder recovered, at least so far as to be able to leave the hospital; though it is probable that in some instances the disease may have returned.

There were thirty-four patients who had the thigh amputated, and one of these had the other leg taken off at the same time below the knee; of this number, nine died. Of twenty-three patients whose legs were amputated below the knee, two having both legs removed, five died; and of the ten who had an arm amputated, six below and four above the elbow, one died.

This goes to confirm the prevailing opinion among surgeons, that amputation of the lower extremities is more often followed by fatal consequences that that of the upper, and that death takes place more frequently after amputation of the thigh, than after that of the leg. More than a quarter of those whose thighs were amputated died, while there was but little more than one death in five among those whose legs were removed below the knee, and only one of the ten whose arms were amputated. This patient too died of delirium tremens. The operation to be sure did not arrest the disease, but apparently contributed nothing to the fatal result.

This table tends also to support the opinions, that patients who undergo amputation for chronic diseases are much more likely to recover than those in whom it is performed in consequence of recent accidents. Of the first class, there were forty-five patients afflicted with various diseases, and of this number all recovered but six; and of the remaining twenty-two, whose limbs were removed on account of recent injuries, no less than ten died; being nearly half of the latter and less than one in seven in the former.

This fact certainly gives support to the opinion, that a state of high health is not favourable to surgical operations; and it also tends to show that death after amputation is not by any means attributable in all cases to the operation alone; for if it were, the proportion of deaths should be as large among one class of patients as among the other.

Dr. Hayward thinks that the result is affected, not only by age and constitution, but by the period at which the operation is performed. He thinks that, in recent cases, it is often done where it might have been avoided—and, in chronic cases, avoided or deferred where it ought to have been done, the patient being allowed to sink into an irremediable condition. He is against operating while the system is under the influence of shock—reaction should occur.

With regard to the ages of the patients operated on, it appears that there were

| | | | | | | | |
|---|---|---|---|---|---|---|---|
| | | | Under 20 | years of age | 13, | of this number | 1 died. |
| Over 20 | and not | exceeding | 30 | " | 31, | " | 8 " |
| " | 30 | " | 40 | " | 9, | " | 3 " |
| " | 40 | " | 50 | " | 10, | " | 2 " |
| " | 50 | " | 60 | " | 3, | " | 1 " |
| | | | Over 70 | " | 1, | " | 0 " |

Whole number, 67. No. of deaths, 15.

